# Elbasvir and grazoprevir for hepatitis C virus genotype 1 infection in people with recent injecting drug use (DARLO‐C): An open‐label, single‐arm, phase 4, multicentre trial

**DOI:** 10.1002/hsr2.151

**Published:** 2020-03-15

**Authors:** Jason Grebely, Phillip Read, Evan B. Cunningham, Martin Weltman, Gail V. Matthews, Adrian Dunlop, Mark Montebello, Marianne Martinello, Rosie Gilliver, Philippa Marks, Tanya L. Applegate, Gregory J. Dore, the DARLO‐C Study Group

**Affiliations:** ^1^ Viral Hepatitis Clinical Research Program The Kirby Institute, UNSW Sydney Sydney Australia; ^2^ Kirketon Road Centre Sydney Australia; ^3^ Department of Gastroenterology and Hepatology Nepean Hospital Penrith Australia; ^4^ Department of Infectious Diseases St Vincent's Hospital Sydney Australia; ^5^ Newcastle Pharmacotherapy Service Newcastle Australia; ^6^ Drug and Alcohol Services South East Sydney Local Health District Sydney Australia

**Keywords:** DAA, drug use, hepatitis C, injecting drug users, PWID, treatment

## Abstract

**Background and Aims:**

Direct‐acting antiviral therapy for hepatitis C virus (HCV) is effective, but few prospective studies among people with ongoing injecting drug use exist. This study evaluated the efficacy of elbasvir/grazoprevir in people with HCV genotype 1/4 (G1/4) infection and recent injecting drug use. An exploratory aim evaluated the feasibility of fingerstick point‐of‐care HCV RNA testing prior to and following treatment.

**Methods:**

DARLO‐C (http://clinicaltrials.gov: NCT02940691) is an open‐label phase 4 trial. Participants were recruited between May 2017 and March 2018 from two drug treatment clinics, two hospital clinics, and one community clinic in Australia. Inclusion criteria included recent injection drug use (previous 6 months) and HCV G1/4 infection. Exclusion criteria included prior HCV treatment and decompensated liver disease. Participants received elbasvir/grazoprevir once‐daily for 12 weeks. The primary endpoint was undetectable HCV RNA 12 weeks post‐treatment (SVR). Fingerstick whole‐blood samples were tested using the Xpert HCV Viral Load Fingerstick (Xpert HCV VL Fingerstick) assay and compared to the Aptima HCV Quant Dx Assay on plasma samples.

**Results:**

Of a planned 150 participants, 32 were enrolled due to slower than anticipated recruitment [median age 46 years, 10 (31%) female, 29 (91%) G1a]. Eighteen (56%) were receiving opioid agonist therapy and 29 (91%) injected in the previous month. Twenty‐six (81%) of 32 completed treatment (lost to follow‐up, n = 5; incarceration, n = 1). There were no virological failures. Twenty‐four (75%, 95% CI 59%‐91%) of 32 achieved SVR. Two participants who completed treatment did not have SVR (loss to follow‐up, n = 1; refused test, n = 1). Among paired samples (n = 36), sensitivity of the Xpert HCV VL Fingerstick assay for HCV RNA detection was 100.0% (95% CI 75.3%‐100.0%) and specificity was 95.7% (95% CI 78.1%‐99.9%).

**Conclusion:**

Elbasvir/grazoprevir is effective among people with HCV G1 with recent injecting drug use. Implementation of point‐of‐care HCV RNA testing was feasible, but the high error rate requires investigation.

## 1 INTRODUCTION

Among the 71 million people with hepatitis C virus (HCV) infection globally,^1^ an estimated 6.1 million people have recently injected drugs (8.5% of all infections).^2^, ^3^ Transmission among people who inject drugs (PWID) is estimated to account for 23% of new infections globally.^4^ As such, it will be difficult to eliminate HCV infection without strategies focused on addressing HCV prevention, testing, linkage to care, and treatment among PWID.

Direct‐acting antiviral therapy is effective in people receiving opioid agonist therapy (OAT) and in people with recent injecting drug use, even in “real‐world” settings.^5^ In the COSTAR study, among people receiving grazoprevir and elbasvir with HCV genotypes 1, 4, or 6 on stable OAT who had never previously received HCV treatment,^6^ the intention‐to‐treat (ITT) SVR was 91%.^6^ However, only 25% had injected drugs within the previous 6 months.^7^ Although many studies have been published on HCV treatment outcomes among people with recent drug use, studies have been limited by being performed at single centres and the heterogeneous study populations considered, particularly with respect to the inclusion of people with recent injecting drug use. Further data on HCV treatment outcomes among people with recent injecting drug use are needed.

Among PWID, poor venous access has been noted as a major barrier to HCV testing and treatment.^8^, ^9^, ^10^, ^11^ PWID with poor venous access may perceive blood collection as distressing due to perceived stigmatisation by healthcare workers (and phlebotomists), inexperience of phlebotomists in collecting blood samples from PWID, and poor access to experienced phlebotomists at services preferred by PWIDs.^12^ Fingerstick whole‐blood collection is acceptable to PWID and is preferred to venepuncture.^10^, ^13^, ^14^ The availability of a fingerstick point‐of‐care HCV RNA test may provide one strategy to address barriers to venous access among PWID.^15^, ^16^, ^17^ The Xpert HCV Viral Load Fingerstick assay (Xpert HCV VL Fingerstick) uses real‐time PCR technology that enables the quantification of HCV RNA levels with 100 μL of capillary whole blood, with results in 1 hour.^15^, ^16^ This assay enables same‐visit HCV diagnosis/treatment, with the potential to enhance linkage to HCV care for PWID. While there are data on the use of the Xpert HCV VL Fingerstick as a tool for screening,^15^, ^16^, ^17^ there are little data on the feasibility of using Xpert HCV VL Fingerstick prior to and during treatment to monitor response to HCV therapy.

This study presents the results of a multicentre, open‐label, phase 4 trial evaluating the efficacy and safety of elbasvir and grazoprevir for 12 weeks in people infected with HCV genotype 1 with recent injecting drug use (previous 6 months). This included an exploratory study to evaluate the feasibility of Xpert HCV VL Fingerstick point‐of‐care testing for HCV RNA quantification prior to, during, and following HCV treatment.

## 2 METHODS

### 2.1 Study design and participants

In this multicentre, open‐label, phase 4 trial, we enrolled participants from five sites in Australia from May 2017 to March 2018 (DARLO‐C, http://clinicaltrials.gov: NCT02940691). We recruited people from two drug treatment clinics, two hospital clinics, and one community clinic.

Participants had to be ≥18 years of age, have chronic HCV genotype 1 or 4 infection (although no one with genotype 4 was enrolled), and have recently injected drugs (self‐reported injecting drug use within 6 months of enrolment). The study protocol was revised in December 2017 to allow the inclusion of people receiving OAT who had not recently injected drugs into the study (although no one fulfilling this inclusion criteria were subsequently enrolled). Participants with HIV infection were eligible for inclusion, but none were enrolled. Participants having previously received any HCV treatment and those with decompensated liver disease were excluded. In the original study protocol, participants required resistance testing to be performed at screening, and participants with any one of the following HCV resistance‐associated NS5A substitutions were excluded: M28L/T/V, Q30H/L/R, L31M, or Y93C/H/N/S. However, given that resistance testing was not broadly available in Australia and not required by national treatment guidelines,^18^ the study protocol was revised in December 2017 to make resistance testing optional. Full eligibility criteria are provided in the study protocol (Supporting Information).

### 2.2 Procedures

Participants with HCV genotype 1 received a fixed‐dose combination tablet that contained 50 mg of elbasvir and 100 mg of grazoprevir orally once‐daily for 12 weeks (funded through the Australian government reimbursement scheme).

Participants attended study visits at screening/enrolment, baseline (treatment initiation), and weeks 4, 8, and 12 (end of treatment) of therapy. Following treatment, participants also attended visits at weeks 16 (SVR4), week 24 (SVR12), and week 36 (SVR24). The study also had visits planned for every 6 months for up to 3 years following the end of treatment.

Enrolment assessments included a venepuncture blood sample [standard of care laboratory and clinical testing (HCV RNA and HCV genotype) and storage for central HCV RNA testing)], Transient Elastography [FibroScan] (where available), and self‐reported behavioural questionnaires. The self‐reported questionnaire was pilot‐tested in collaboration with community‐based drug user organizations and used in other studies of HCV therapy among PWIDs.^19^, ^20^, ^21^ Assessments during treatment included physical examinations, measurements of HCV RNA levels (performed at local laboratories), and standard laboratory testing (liver function tests, full blood count, and biochemistry). All adverse events were recorded and graded according to the Medical Dictionary for Regulatory Activities, MedDRA.

As part of an exploratory study to assess the feasibility of fingerstick point‐of‐care HCV RNA testing, participants were offered Xpert HCV Viral Load Fingerstick (Xpert HCV VL Fingerstick) testing at all study visits at four sites. The study protocol was revised in December 2017 to reduce the number of visits at which fingerstick HCV RNA testing was performed (removing testing at week 8 and end of treatment).

A capillary whole‐blood sample was collected from participants via a fingerstick (Safety Lancet, Super Blade [Order Number 85.1018], Sarstedt, Nümbrecht, Germany) using procedures recommended by the WHO^22^ and collected into a 100‐μL minivette collection tube (Minivette POCT 100 μL K3E [Order number 17.2113.101], Sarstedt, Nümbrecht, Germany). Immediately following collection, 100 μL of capillary whole blood was placed directly into the Xpert HCV VL Fingerstick assay prototype cartridge (research use only, lower limit of quantification of 100 IU/mL; Cepheid, Sunnyvale) for onsite HCV RNA testing. The cartridge was loaded into the GeneXpert instrument which uses real‐time PCR (qPCR) technology that enables the quantification of HCV RNA levels.^23^ The time to result for Xpert HCV VL Fingerstick testing is 58 minutes.

All Xpert HCV Viral Load assay testing were performed on a clinic‐based GeneXpert R2 6‐colour, 4 module machine (GXIV‐4‐L System, 900‐0513, GeneXpert Dx software v4.6a; Cepheid, Sunnyvale) operated as per the manufacturer's instructions.^23^ Participants were not provided the result of their Xpert HCV test results, given that the Xpert HCV Viral Load assay is not approved in Australia.

HCV RNA levels for evaluation of the primary endpoint (SVR12) were measured on stored plasma samples tested centrally with the Aptima HCV Quant Dx Assay (Hologic, Cat. No. PRD‐03705; lower limit of detection <10 IU/mL; lower limit of quantification of <25 IU/mL). Central HCV RNA testing was performed on samples collected at baseline, week 12 (end of treatment), week 24 (SVR12), and at most recent available. HCV genotype/subtype was determined by sequencing the NS5A and NS5B regions using Sanger sequencing.

Participants completed a self‐administered questionnaire at screening/enrolment, at baseline (treatment commencement), every fourth week during treatment, and at 12 weeks post‐treatment (participants received the equivalent of AUD$20 reimbursement for their time). The questionnaires collected information on demographics (age, gender, employment status, education level, housing status), drug/alcohol use, injecting risk behaviours, and drug treatment. Stable housing was defined as living in a rented or owned house or flat. Alcohol consumption was evaluated by the Alcohol Use Disorders Identification Test‐Consumption (AUDIT‐C), derived from the first three questions of the full AUDIT [scores ≥3 (women) and ≥4 (men) indicate hazardous consumption or active alcohol use disorders].^24^

Stage of liver fibrosis was assessed by liver stiffness measurement (Transient Elastography [FibroScan]) or AST‐to‐Platelet Ratio Index (APRI). For liver stiffness measurements, the chosen cut‐offs for significant liver fibrosis was 7.1 kPa and was 12.5 kPa for cirrhosis.^25^ APRI was calculated using aspartate aminotransferase (AST) and platelet count: [(AST [U/L]/upper limit of normal)/platelet count (10^9^/L)] × 100. APRI >1.0 and >2.0 defined significant liver fibrosis and cirrhosis, respectively.

### 2.3 Outcomes

The primary efficacy endpoint was the proportion of participants with a SVR12, defined as an HCV RNA level below the limit of quantification 12 weeks after the end of treatment in all participants who received at least one dose of study medication (ITT population). When HCV RNA had not been assessed at SVR12, the result of the next available HCV RNA assessment was used to calculate SVR. Participants with no result at or following the SVR12 visit were considered to not have had an SVR. In addition, a post hoc modified ITT analysis was performed excluding participants with a missing SVR12 test. Secondary endpoints included treatment completion, treatment adherence, end of treatment response (ETR, negative HCV RNA at the end of treatment), severe adverse events, and treatment discontinuations because of adverse events.

### 2.4 Statistical analysis

The primary aim of this study was to evaluate the efficacy of elbasvir and grazoprevir for 12 weeks in participants infected with HCV genotype 1 with recent injecting drug use (previous 6 months).

A total of 150 participants were planned for enrolment and evaluation as the ITT population. Assuming an overall SVR of 90% (135 of 150), the 95% confidence interval (95% CI) around this estimate would be 84% to 94%. However, due to slower than anticipated enrolment as a result of the availability of pan‐genotypic regimens in Australia leading to reduced prescribing of the elbasvir and grazoprevir regimen, recruitment was closed prematurely (n = 32). Assuming an overall SVR of 90%, the 95% CI around this estimate (calculated using Clopper‐Pearson binomial confidence intervals) would be expected to be 75% to 98%.

We used the Clopper‐Pearson method to calculate point estimates and two‐sided 95% exact confidence intervals for the proportion with SVR overall, as well as according to HCV genotype, and various subgroups. Factors hypothesized to be associated with SVR were age (stratified by median), gender, current OAT at baseline, recent (previous month) injecting drug use at baseline, and frequency of injecting at baseline (none, less than daily, daily, or greater).^5^, ^26^

The sensitivity and specificity of the Xpert HCV Viral Load Fingerstick assay for detection of HCV RNA in plasma samples collected via venepuncture and capillary whole‐blood samples collected by fingerstick was assessed using both detectable and quantifiable thresholds (limit of quantification >100 IU/mL Xpert HCV VL Fingerstick) compared to Aptima HCV Quant Dx assay in plasma as the reference standard (limit of quantification >10 IU/mL). Any discordant results were included in all calculations of sensitivity and specificity. All data are reported in log_10_ units.

For all analyses, statistically significant differences were assessed at a 0.05 level; *P*‐values were two‐sided. All analyses were performed using Stata v12.0 (StataCorp, College Station, Texas).

### 2.5 Ethical considerations

All participants provided written informed consent before study procedures. The study protocol was approved by St. Vincent's Hospital, Sydney Human Research Ethics Committee (primary study committee) and was conducted according to the Declaration of Helsinki and International Conference on Harmonization Good Clinical Practice (ICH/GCP) guidelines. The study was registered with http://clinicaltrials.gov registry (NCT02498015).

### 2.6 Role of the funding source

The study was funded by a research grant from Merck/MSD. The funder had no role in the study design, data collection, analysis, interpretation of the results, the writing of the report, or the decision to submit the report for publication. J.G., E.C., and G.D. had access to the raw data. The sponsor (The Kirby Institute, UNSW Sydney) designed the study, collected the data, managed study samples, monitored study conduct, and performed the statistical analysis. J.G. and G.D. were responsible for the decision to submit for publication.

## 3 RESULTS

### 3.1 Participant characteristics

**Figure 1:**
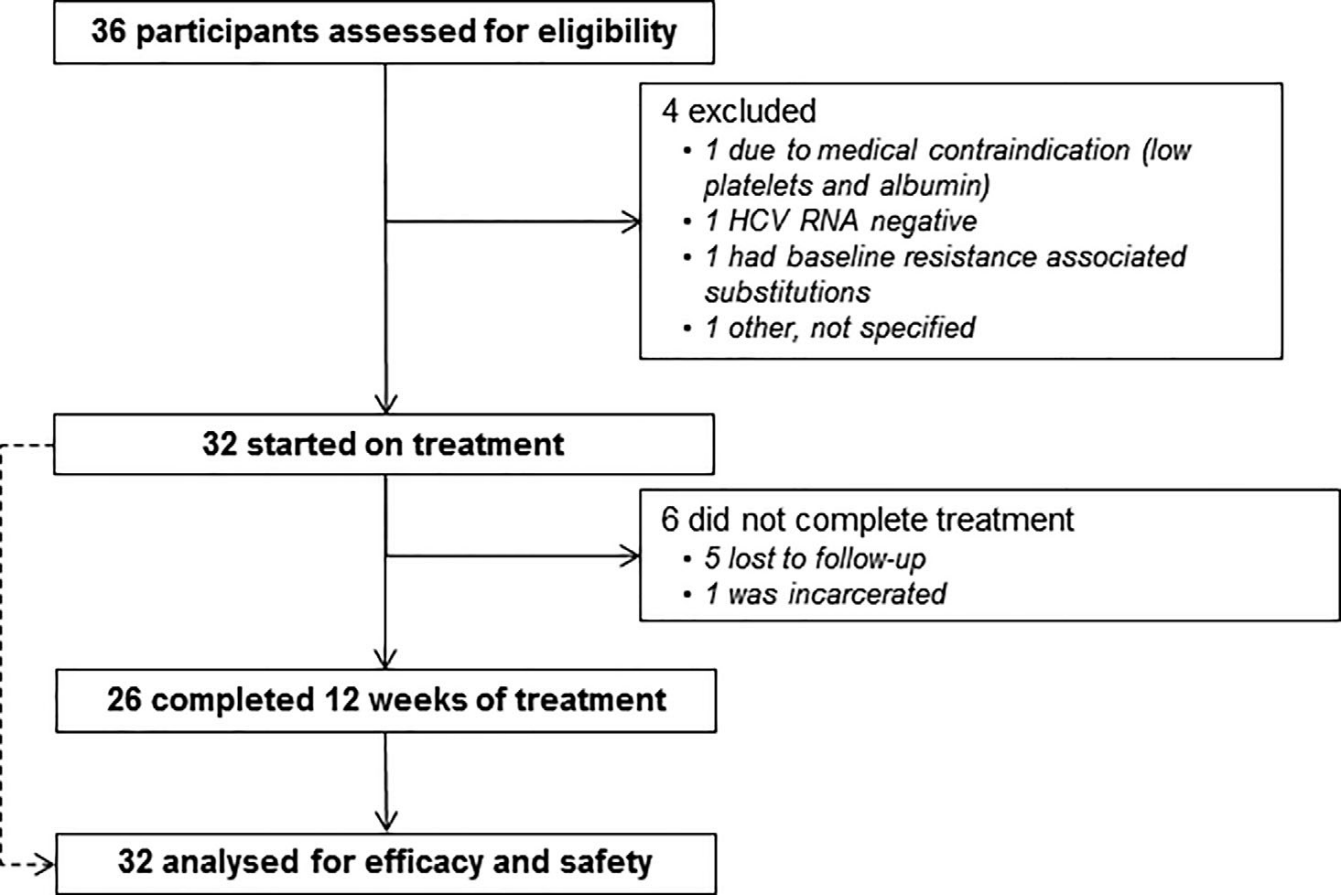
Study profile

Of 36 participants screened, 32 were enrolled and received at least one dose of study medication (ITT population, Figure 1, Table 1). Most participants (91%, 29 of 32) had genotype 1a. The median age was 46 years, 31% (10 of 32) were female, and 6% (2 of 32) had cirrhosis.

**Table 1:** Baseline characteristics

	Elbasvir and grazoprevir for 12 weeks (n = 32)
Age (years)	46 (38‐52)
Female gender	10 (31%)
High school or higher education	6 (19%)
Unstable housing^a^	12 (38%)
Income	
°Full‐time employment	0 (0)
°Part‐time employment	1 (3%)
°Disability/social services	30 (94%)
°Other	1 (3%)
Any non‐injecting drug use in the previous month	11 (34%)
Any injecting drug use in the previous month	29 (91%)
°Heroin	16 (50%)
°Cocaine	0 (0)
°Methamphetamines	21 (66%)
°Other opioids	8 (25%)
Injecting drug use frequency in the previous month	
°Never	5 (16%)
°<daily	18 (56%)
°≥daily	9 (28%)
Any alcohol use in the previous month	24 (75%)
Hazardous alcohol use in the previous month	23 (72%)
History of OAT	23 (72%)
Current OAT	18 (56%)
°Methadone	15 (47%)
°Buprenorphine	2 (6%)
°Buprenorphine/naloxone	3 (9%)
OAT and had injected in previous month (baseline)	
°No OAT, no recent injecting	2 (6%)
°No OAT, recent injecting	12 (38%)
°OAT, no recent injecting	3 (9%)
°OAT, recent injecting	15 (47%)
HCV genotype	
°1a	29 (91%)
°1b	3 (9%)
HCV RNA, log IU/mL	5.0 (5.5‐6.4)
Alanine transaminase, IU/L^b^	43 (31‐75)
Stage of liver disease	
°No or mild fibrosis (F0‐F1)^c^	23 (72%)
°Moderate or advanced fibrosis (F2‐F3)^c^	7 (22%)
°Cirrhosis (F4)^c^	2 (6%)

*Note*: Data are n (%), or median (IQR). Data were unavailable for two participants.

Abbreviations: HCV, hepatitis C virus; OAT, opioid agonist therapy.

a Stable housing was defined as a rented or privately owned house or flat.

b Data were unavailable for seven participants.

c F0‐F1 < 7·1 kPa, F2–F3 7·1‐12·49 kPa, F4 ≥ 12·5 kPa.

**Table 2:** Sensitivity and specificity of the Xpert HCV VL Fingerstick assay for HCV RNA quantification compared to the Aptima HCV Quant Dx assay

Xpert HCV VL Fingerstick	Aptima HCV Quant Dx		
	Number detectable	Number undetectable	Total number
Number detectable	13	1	14
Number undetectable	0	22	22
Total number	13	23	36

Abbreviation: HCV, hepatitis C virus.

**Figure 2:**
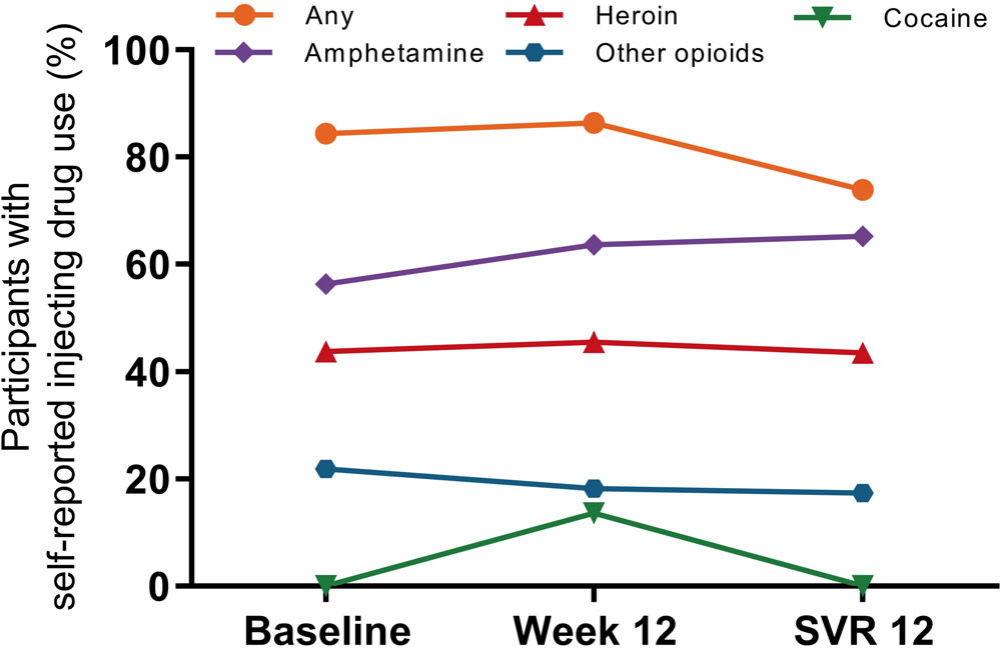
Self‐reported injecting drug use during therapy

At baseline, 91% (29 of 32) had injected drugs in the previous month, 28% (9 of 32) had injected drugs ≥daily in the previous month, and 56% (18 of 32) were receiving OAT (Table 1). The most commonly injected drugs were methamphetamines (21 of 32; 66%), heroin (16 of 32; 50%), and other opioids (8 of 32; 25%). As shown in Figure 2, drug use remained relatively stable during treatment.

### 3.2 Overall HCV treatment completion, adherence, and outcomes

Among all participants enrolled, 26 (81%) of 32 completed treatment. Of the six participants who did not complete treatment (Figure 1), five discontinued due to loss to follow‐up [at weeks 4, 8 (n = 2), and 12 (n = 2)], and one discontinued due to incarceration (at week 4).

In ITT analysis, 24 (75%, 95% CI: 59%, 91%) of 32 had an ETR and 24 (75%, 95% CI: 59%, 91%) of 32 had an SVR. Among participants who completed treatment but did not have an SVR (n = 2), reasons for not achieving an SVR included an inability to obtain a blood sample post‐treatment and refusing to have an SVR test. There were no virologic relapses. In a modified ITT analysis (excluding people without an SVR test), SVR was 100% (24 of 24 with available testing). No reinfections were observed (eight person‐years of follow‐up). The proportion with SVR stratified by key characteristics is shown in Table S1.

### 3.3 Safety

A total of five (16%) of 32 participants experienced a serious adverse event that required hospitalisation. These events were considered not to be related to the study drugs and included drug‐induced psychosis, laceration of arm, schizoaffective disorder, suicidal ideation, and psychosis. There were no deaths.

### 3.4 Fingerstick point‐of‐care HCV RNA testing for on‐treatment monitoring

**Figure 3:**
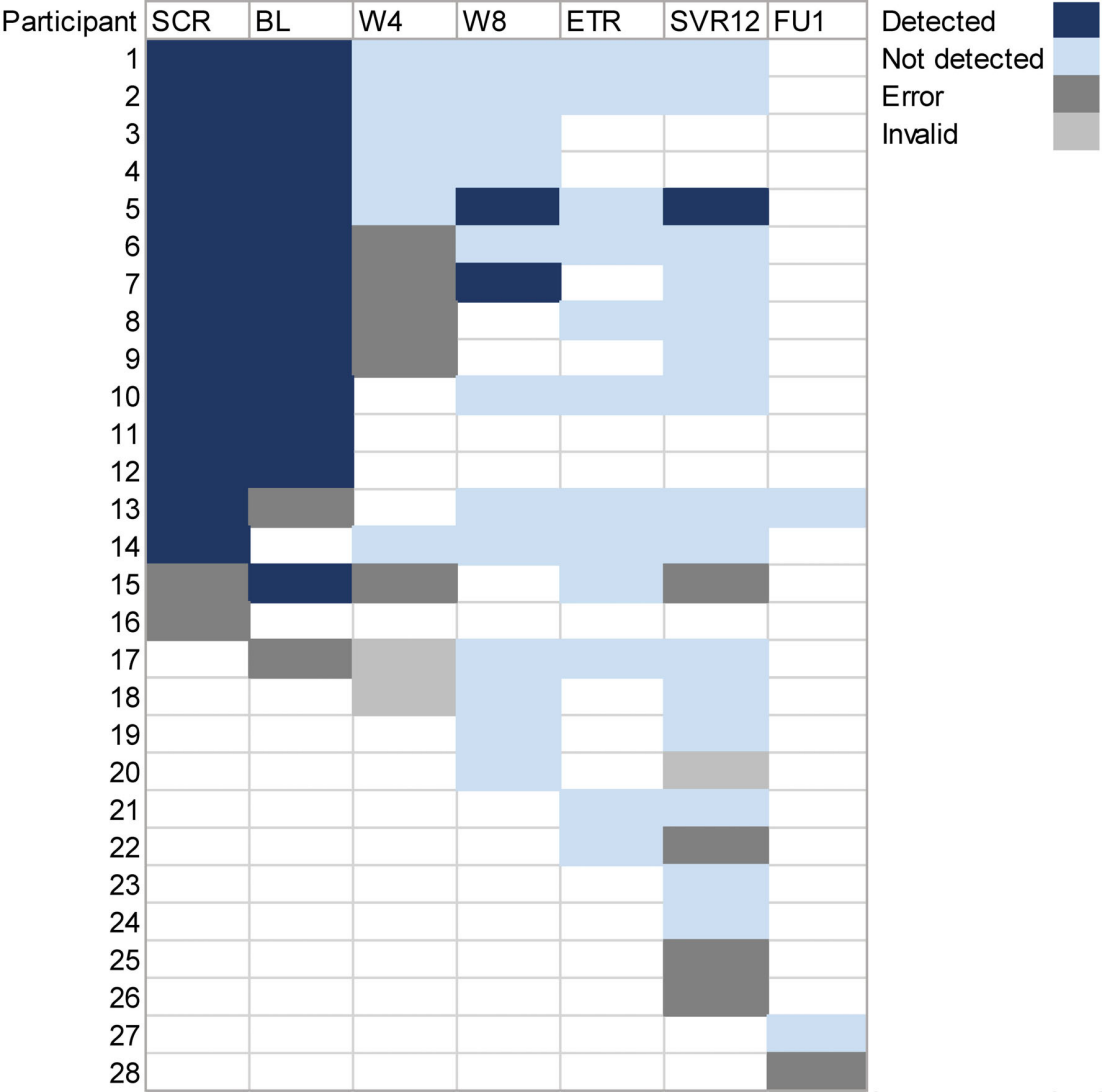
Fingerstick point‐of‐care HCV RNA testing for on‐treatment monitoring with the Xpert HCV viral load Fingerstick assay. SCR, screening; BL, baseline; W4, week 4; W8, week 8; ETR, end of treatment; SVR12, sustained virologic response; and FU1, follow‐up visit one. Participants 17 to 28 only had available testing at the given time‐points, given the later implementation of Xpert HCV viral load Fingerstick testing

Overall, 28 (88%) of 32 participants had at least one available fingerstick sample (94 total samples; Figure 3). Reasons for not having a valid result (n = 17) included errors reported by the Xpert equipment (n = 14, 15%) and invalid results due to the internal controls being out of range (n = 3, 3%). Samples were available at screening/baseline for 17 (53%) participants, week 4 for 13 (41%) participants, week 8 for 14 (44%) participants, end of treatment for 12 (38%) participants, and SVR12 or greater in 23 (72%) participants. Among 36 paired samples available for a comparison of the Xpert HCV VL Fingerstick and Aptima HCV Quant Dx assay, the sensitivity of the Xpert HCV VL Fingerstick assay for HCV RNA detection in capillary whole‐blood samples collected by fingerstick was 100.0% (95% CI 75.3%‐100.0%) and specificity was 95.7% (95% CI 78.1%‐99.9%) (Table 2).

## 4 DISCUSSION

This study describes the results of a multicentre, open‐label, phase 4 trial evaluating the efficacy and safety of elbasvir and grazoprevir for 12 weeks in people infected with HCV genotype 1 with recent injecting drug use. The proportion with SVR was 75%. There were no virologic failures, and failure to achieve an SVR was primarily due to loss to follow‐up. This study also suggested a high sensitivity and specificity for HCV RNA detection with the Xpert HCV VL Fingerstick point‐of‐care assay compared to the Aptima HCV Quant Dx Assay, but a high error rate was observed. This study provides additional data to contribute to clinical guidelines for treatment of HCV among people who have recently injected drugs. Further work is needed to evaluate the accuracy, impact, and cost‐effectiveness of fingerstick point‐of‐care testing for detection of SVR and post‐treatment monitoring for HCV reinfection.

The overall proportion with SVR of 75% is lower than the weighted mean SVR of 87% in a systematic review of direct‐acting antiviral (DAA) therapy among people who recently injected drugs.^5^ In this systematic review, a meta‐regression analysis was also performed^5^ and “real‐world” observational studies were associated with a lower SVR compared to clinical trials. The lower SVR was explained by a higher proportion of participants lost to follow‐up in “real‐world” observational studies compared to clinical trials.^5^ This is consistent with the results in the current study demonstrating that only 81% of participants completed treatment and lost to follow‐up was the primary reason for not achieving an SVR. In fact, there were no cases of virological failure in this study. The lower treatment completion and SVR observed in this study is consistent with a “real‐world” study of DAA treatment among people who have recently injected drugs in Sydney, Australia.^27^ In this study by Read et al, one‐third of people elected to receive daily or weekly enhanced adherence support, resulting in equivalent follow‐up for SVR testing and SVR to those who did not receive enhanced adherence support.^27^ It is interesting that those who received enhanced treatment support in this study were more often homeless, identified as Aboriginal, had a mental health diagnosis, and ≥daily injecting drug use.^27^ Among those who discontinued therapy early, two‐thirds (4 of 6) discontinued after week 8 of therapy. Shorter durations of HCV therapy (eg, <8 weeks) could be explored for populations who might be at higher risk of early discontinuation, such as people with ongoing injecting drug use. Collectively, these data suggest that strategies should be explored to improve retention and facilitate HCV treatment completion, particularly among marginalized PWIDs who may require enhanced support during therapy.

Injecting and non‐injecting drug use was stable prior to and during HCV therapy, consistent with results from studies of interferon‐based^28^, ^29^, ^30^ and DAA‐based therapies.^6^, ^19^, ^20^, ^31^, ^32^ SVR was high among people with ≥daily injecting drug use, consistent with previous literature.^5^ A strength of this study is that it represents a population of people with more recent injecting drug use, with 91% of people enrolled stating that they had injected drugs in the previous month. Many of the previous studies performed to date have included study populations with heterogeneous definitions of injecting drug use (eg, included people with and without recent injecting drug use) or broad definitions of recent injecting drug use (eg, injecting in the previous year).

The sensitivity of the Xpert HCV VL Fingerstick test for HCV RNA quantification by fingerstick whole blood prior to, during, and following DAA therapy was 100%, while the specificity was 95.7%. This is consistent with previous studies demonstrating a good sensitivity and specificity of the Xpert HCV VL Fingerstick test from capillary whole‐blood samples collected from fingerstick and dried blood spots for the diagnosis of HCV infection.^15^, ^16^, ^33^ In this reduced subset of samples, there was only one discrepant sample that was detected by the Xpert HCV VL Fingerstick test but not detectable with Aptima HCV Quant Dx Assay. Among the samples tested, there were 18% of samples that failed to provide a result on the Xpert HCV VL Fingerstick assay due to errors (15%, sampling issue) and invalid results (3%, internal control out of range mostly due to early prototype). During the study, we discovered that we had received a batch of cartridges that had been damaged (based on the presence of crystallized reagents) during manufacturing or shipping. This may partially explain the high failure rate. Some errors were also due to the collection of inadequate sample volumes. As such, the reported sensitivity and specificity reflects the optimal performance of the assay in the absence of errors. The current evaluation was conducted on an early prototype of the HCV VL Fingerstick cartridge and the availability of new versions of the cartridge has reduced the number of errors and repeats tests required.^34^ In a recent study performed by our group, among 1386 participants with Xpert HCV VL Fingerstick testing performed by a single technician, only 2.9% samples failed to provide a result (1.5% were errors due to low sample volume, 1.3% invalid results due to the internal controls being out of range, and 0.1% other).^34^ In the current study, improvements in machine operation were observed with increased operator experience. Collectively, these data illustrate the importance of appropriate training of machine operators and quality assurance programs to ensure valid results. Further evaluation of the most recent prototype of the HCV VL Fingerstick cartridge is needed to ensure that errors and invalid results do not preclude broader implementation of this technology. However, given that venous access can be problematic among PWID, there is a greater acceptability of fingerstick HCV RNA testing compared to venepuncture.^10^ Fingerstick HCV RNA testing could provide a useful tool for confirming viral cure and monitoring for HCV reinfection following successful DAA therapy. Further research is needed to understand how Xpert HCV VL Fingerstick performs in the setting of monitoring HCV treatment outcomes and HCV reinfection following successful treatment.

This study has limitations. Given that participants were recruited from HCV clinics in tertiary hospitals, drug treatment clinics, and community health centres experienced in providing HCV care, the results of this study may not be generalizable to all populations of PWID and to all HCV treatment settings. Further, participants received a AUD$20 incentive at all study‐related visits, which may have provided additional incentive to return for follow‐up and led to an improved retention in the study. It should also be noted that this study was discontinued prematurely due to slower than anticipated enrolment, related to the broader access to DAA therapy following the availability of pangenotypic HCV therapies with once‐daily dosing. As such, this limited the number of participants recruited into the study and resulted in a smaller‐than anticipated sample size and limited the power to investigate factors associated with SVR (which is why these results are presented only descriptively). Although the availability of pangenotypic HCV therapies has limited the use of elbasvir and grazoprevir, it is still used in some countries, including in the United Kingdom and the United States. Irrespective of whether this regimen remains broadly used, the findings of this study are still applicable for informing HCV clinical management among PWIDs. Xpert HCV VL Fingerstick testing was introduced during the conduct of the study, so not all participants had available samples at all time‐points. Further, paired HCV RNA testing was not performed at all study visits, so this reduced the sample size for analyses of sensitivity and specificity and may have resulted in a biased subset of samples. Lastly, given the early termination of the study, there was limited follow‐up after successful DAA therapy (eight person‐years of follow‐up), impacting an evaluation of HCV reinfection.

This study evaluated HCV treatment outcomes following elbasvir and grazoprevir therapy among people who have recently injected drugs with HCV genotype 1 infection, the large majority of whom had injected in the previous month. This study also provides a proof‐of‐concept for the evaluation of the Xpert HCV VL Fingerstick assay prior to and following DAA treatment among people who have recently injected drugs. A major limitation of this study was the small sample size due to the premature discontinuation of the study because of slower than anticipated enrolment, thereby limiting the conclusions that can be drawn from these data. Further research is needed to evaluate the potential of Xpert HCV VL Fingerstick testing and other testing modalities (eg, dried blood spot testing) as potential strategies to simplify and enhance HCV testing, linkage to care, and treatment. This includes understanding the barriers to and acceptability of uptake and integration of point‐of‐care HCV RNA testing in different services for PWIDs. As part of a cluster randomized trial, the TEMPO study (http://clinicaltrials.gov, NCT04014179) will evaluate the effect of dried blood spot or point‐of‐care HCV RNA testing on uptake of HCV treatment among people with recent injecting drugs attending primary needle syringe programs in Australia. Achieving elimination of HCV infection as a major public health threat among PWID will require further research evaluating novel strategies to enhance linkage to testing and treatment and ensure that these interventions are delivered at scale to achieve maximal public health benefit.

## CONFLICT OF INTEREST

Dr Grebely reports grants from Abbvie during the conduct of the study; and grants and personal fees from Abbvie, grants and personal fees from Gilead Sciences, grants and personal fees from Merck, grants and personal fees from Cepheid, outside the submitted work. Dr Read reports research grants from Gilead Sciences, and personal fees from Gilead and Merck outside the submitted work. Dr Dore reports grants from Gilead, grants from Abbvie, grants from Merck, grants from Bristol‐Myers Squibb, personal fees from Gilead, personal fees from Abbvie, personal fees from Merck, personal fees from Bristol‐Myers Squibb, non‐financial support from Gilead, non‐financial support from Abbvie, non‐financial support from Merck, non‐financial support from Bristol‐Myers Squibb, outside the submitted work. The interests declared did not affect the design, conduct, or reporting of this study.

## AUTHOR CONTRIBUTIONS

Conceptualization: Jason Grebely, Gregory Dore, Philippa Marks, and Phillip Read

Data Curation: Evan Cunningham

Formal Analysis: Evan Cunningham

Funding acquisition: Jason Grebely and Gregory Dore

Investigation: Phillip Read, Martin Weltman, Gail V. Matthews, Adrian Dunlop, Mark Montebello, Marianne Martinello, Rosie Gilliver, and Gregory Dore

Project Administration: Philippa Marks

Supervision: Jason Grebely and Gregory Dore

Writing – Original Draft Preparation: Jason Grebely

Writing – Review & Editing: Phillip Read, Evan Cunningham, Martin Weltman, Gail V. Matthews, Adrian Dunlop, Mark Montebello, Marianne Martinello, Rosie Gilliver, Tanya L Applegate, and Gregory Dore

## TRANSPARENCY STATEMENT

The corresponding author (J.G.) affirms that this manuscript is an honest, accurate, and transparent account of the study being reported; that no important aspects of the study have been omitted; and that any discrepancies from the study as planned (and, if relevant, registered) have been explained.

## DATA AVAILABILITY STATEMENT

The individual deidentified participant data (including data dictionaries) that support the findings of this study (text, tables, figures, and appendices) are available from the corresponding author upon reasonable request. The study protocol is included as Supporting Information, and the study is registered at http://clinicaltrials.gov: NCT02940691. Data may be requested from the corresponding author by email (with an appropriate plan for the use of data) by investigators wishing to perform individual‐level meta analyses that has been approved by an independent review committee identified for this purpose. Proposals may be submitted immediately following publication.

## Supporting information


**Table S1** SVR12 (95% CIs), stratified by key characteristics
