# Supplementary material for: Elbasvir and grazoprevir for hepatitis C virus genotype 1 infection in people with recent injecting drug use (DARLO‐C): An open‐label, single‐arm, phase 4, multicentre trial
Source: Health Sci Rep. 2020 Mar 15;3(2):e151. doi: 10.1002/hsr2.151 (PMC7136479; doi:10.1002/hsr2.151)
Supplement: Supplementary file 1 — Table S1 SVR12 (95% CIs), stratified by key characteristics [file HSR2-3-e151-s001.docx]

**Supplementary Table 1. SVR12 (95% CIs), stratified by key characteristics**

|  | n | SVR | no SVR | **SVR %**  **(95% CI)** |
| --- | --- | --- | --- | --- |
| Age |  |  |  |  |
| ≤46 years | 16 | 13 | 3 | 81 (54, 96) |
| >46 years | 16 | 11 | 5 | 69 (41, 89) |
| Gender |  |  |  |  |
| Male | 22 | 16 | 6 | 73 (50, 89) |
| Female | 10 | 8 | 2 | 80 (44, 97) |
| Current opioid agonist therapy at baseline |  |  |  |  |
| No | 14 | 10 | 4 | 71 (42, 92) |
| Yes | 18 | 14 | 4 | 78 (52, 94) |
| Recent injecting at baseline (previous month) |  |  |  |  |
| No | 3 | 2 | 1 | 67 (9-99) |
| Yes | 29 | 22 | 7 | 76 (56-90) |
| Frequency of injecting at baseline (previous month) |  |  |  |  |
| None | 3 | 2 | 1 | 67 (9-99) |
| Less than daily | 18 | 13 | 5 | 72 (47-90) |
| Daily or greater | 9 | 8 | 1 | 89 (52-100) |
